# Supplementary material for: DeepBindPoc: a deep learning method to rank ligand binding pockets using molecular vector representation
Source: PeerJ. 2020 Apr 6;8:e8864. doi: 10.7717/peerj.8864 (PMC7144620; doi:10.7717/peerj.8864)
Supplement: Supplemental Information 1 — The performance of DeepBindPoc_native model which training by native pocket as positive (Training B). The normalization strategy is based on Training dataset. The Training B, Validation B, and Testing B below are using native as positive. The Training A, Validation A, and Test A are the generated dataset with the near-native as positive dataset. [file peerj-08-8864-s001.docx]

**Table S1.** The performance of DeepBindPoc_native model which training by native pocket as positive (Training B). The normalization strategy is based on Training dataset. The Training B, Validation B, and Testing B below are using native as positive. The Training A, Validation A, and Test A are the generated dataset with the near-native as positive dataset.

| **Dataset** | **AUC** | **Acc** | **TPR** | **Pre** | **MCC** | **Pos_size** | **Neg_size** |
| --- | --- | --- | --- | --- | --- | --- | --- |
| Training B | 1.00 | 0.97 | 0.94 | 1.00 | 0.94 | 6000*3 | 18000 |
| Validation B | 1.00 | 0.97 | 0.94 | 1.00 | 0.94 | 1000 | 1000 |
| Test B | 1.00 | 0.97 | 0.93 | 1.00 | 0.94 | 7491 | 5822 |
| Training A | 1.00 | 0.96 | 0.93 | 1.00 | 0.93 | 6000*3 | 18000 |
| Validation A | 0.92 | 0.60 | 0.19 | 1.00 | 0.32 | 1000 | 1000 |
| Test A | 0.92 | 0.59 | 0.18 | 0.99 | 0.31 | 677 | 5822 |
