# Supplementary material for: DeepBindPoc: a deep learning method to rank ligand binding pockets using molecular vector representation
Source: PeerJ. 2020 Apr 6;8:e8864. doi: 10.7717/peerj.8864 (PMC7144620; doi:10.7717/peerj.8864)
Supplement: Supplemental Information 2 — Demonstrate the predicted value of DeepBindPoc on the decoys of extra test C (6QTN and 5ZG2). The fpocket prediction value was given for comparison. The normalized strategy is based on the training set. The pocket_A and pocket_B are top predicted pocket of 6QTN by DeepBindPoc score for GTP and ACP, respectively. The pocket_C and pocket_D are the top predicted pockets of 5ZG2 by DeepBindPoc score for ZKI and 9C0, respectively. [file peerj-08-8864-s002.docx]

**Table S2.** Demonstrate the predicted value of DeepBindPoc on the decoys of extra test C (6QTN and 5ZG2). The fpocket prediction value was given for comparison. The normalized strategy is based on the training set. The pocket_A and pocket_B are top predicted pocket of 6QTN by DeepBindPoc score for GTP and ACP, respectively. The pocket_C and pocket_D are the top predicted pockets of 5ZG2 by DeepBindPoc score for ZKI and 9C0, respectively.

| **Pocket_A** | **DeepBindPoc Score（GTP as ligand）** | **Fpocket Score** | **Pocket_B** | **DeepBindPoc Score (ACP as ligand)** | **Fpocket Score** |
| --- | --- | --- | --- | --- | --- |
| **6QTN case** | | | | | |
| 6QTN_poc47 | 0.78 | -0.10 | 6QTN_poc52 | 0.98 | -0.37 |
| 6QTN_poc1 | 0.70 | 0.42 | 6QTN_poc47 | 0.97 | -0.10 |
| 6QTN_poc52 | 0.50 | -0.37 | 6QTN_poc41 | 0.92 | -0.07 |
| 6QTN_poc17 | 0.20 | 0.04 | 6QTN_poc32 | 0.89 | -0.03 |
| 6QTN_poc13 | 0.19 | 0.09 | 6QTN_poc1 | 0.74 | 0.42 |
| 6QTN_poc35 | 0.17 | -0.04 | 6QTN_poc13 | 0.67 | 0.09 |
| 6QTN_poc2 | 0.02 | 0.24 | 6QTN_poc7 | 0.52 | 0.15 |
| 6QTN_poc7 | 0.02 | 0.15 | 6QTN_poc35 | 0.48 | -0.04 |
| 6QTN_poc19 | 0.01 | 0.03 | 6QTN_poc2 | 0.41 | 0.24 |
| 6QTN_poc20 | 0.01 | 0.03 | 6QTN_poc17 | 0.39 | 0.04 |
| \| **Pocket_C** \| **DeepBindPoc Score（ZKI as ligand）** \| **Fpocket Score** \| **Pocket_D** \| **DeepBindPoc Score(9C0 as ligand)** \| **Fpocket Score** \| \| --- \| --- \| --- \| --- \| --- \| --- \| | | | | | |
| **5ZG2 case** | | | | | |
| 5ZG2_poc13 | 1.00 | -0.05 | 5ZG2_poc19 | 1.00 | -0.16 |
| 5ZG2_poc19 | 1.00 | -0.16 | 5ZG2_poc13 | 1.00 | -0.05 |
| 5ZG2_poc22 | 1.00 | -0.22 | 5ZG2_poc22 | 0.99 | -0.22 |
| 5ZG2_poc20 | 0.99 | -0.17 | 5ZG2_poc23 | 0.10 | -0.39 |
| 5ZG2_poc2 | 0.99 | 0.10 | 5ZG2_poc20 | 0.03 | -0.17 |
| 5ZG2_poc23 | 0.99 | -0.39 | 5ZG2_poc7 | 0.02 | 0.00 |
| 5ZG2_poc8 | 0.98 | -0.01 | 5ZG2_poc5 | 0.02 | 0.00 |
| 5ZG2_poc16 | 0.94 | -0.06 | 5ZG2_poc16 | 0.01 | -0.06 |
| 5ZG2_poc7 | 0.93 | 0.00 | 5ZG2_poc10 | 0.00 | -0.02 |
| 5ZG2_poc5 | 0.93 | 0.00 | 5ZG2_poc2 | 0.00 | 0.10 |
