# Supplementary figures and images for: DeepBindPoc: a deep learning method to rank ligand binding pockets using molecular vector representation
Source: PeerJ. 2020 Apr 6;8:e8864. doi: 10.7717/peerj.8864 (PMC7144620; doi:10.7717/peerj.8864)

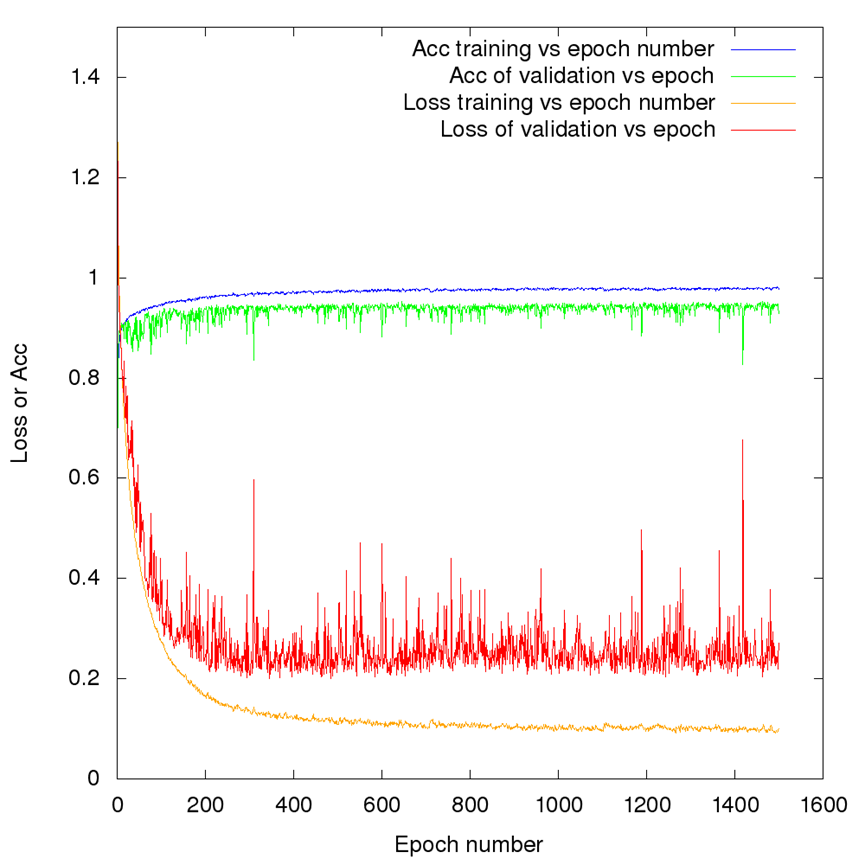

Supplement: Supplemental Information 3 [file peerj-08-8864-s003.png]

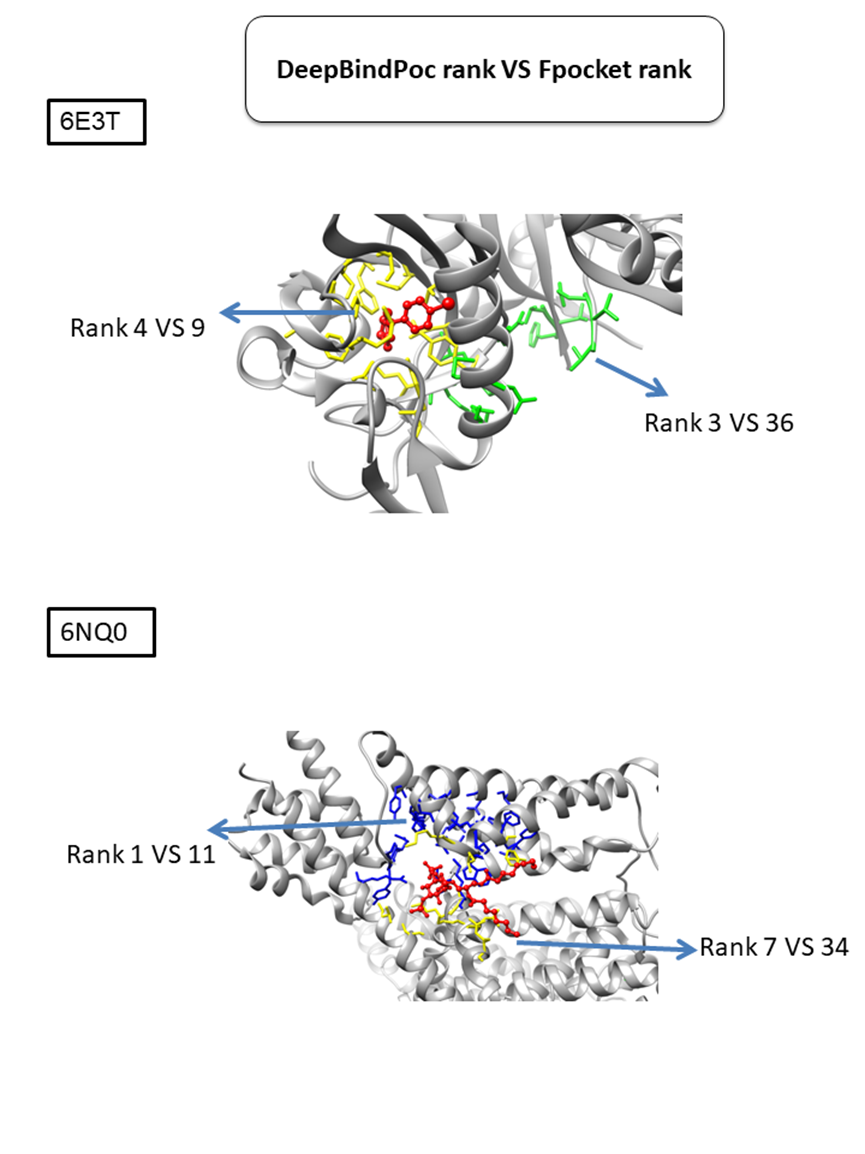

Supplement: Supplemental Information 4 — Some of the false-positive pocket in extra data set predicted by DeepBindPoc is still near the native. [file peerj-08-8864-s004.png]

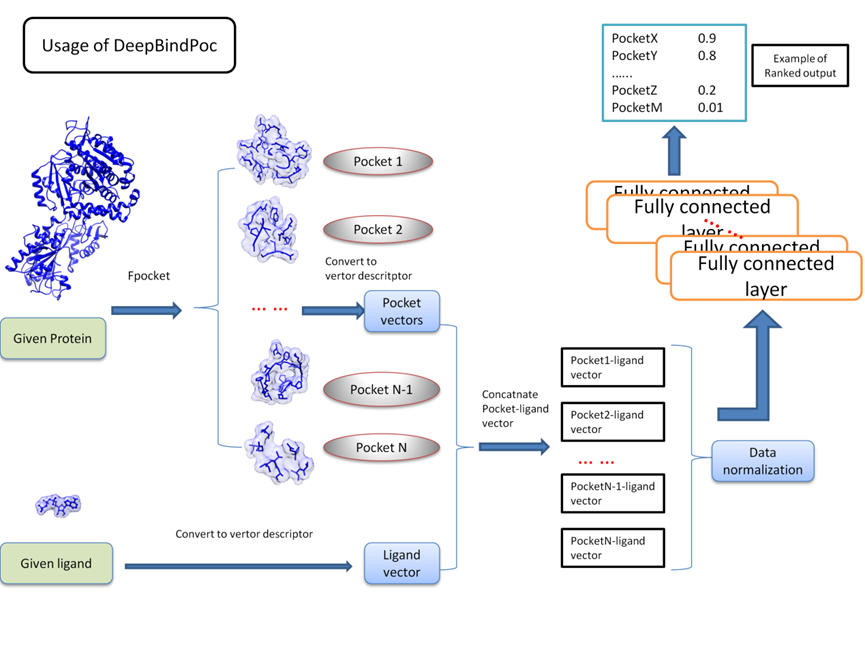

Supplement: Supplemental Information 5 [file peerj-08-8864-s005.png]
